# Fever and Painful Skin Lesions: When to Consider Sweet's Syndrome

**DOI:** 10.7759/cureus.117840

**Published:** 2026-10-13

**Authors:** Mariana Marques, Joana Couto, Joana Urbano, José Miguel Gonçalves, José Miguel Sá, Manuel Veiga

**Affiliations:** 1 Internal Medicine, Hospital Vila Franca de Xira, Vila Franca de Xira, PRT; 2 Internal Medicine, Unidade Local de Saúde do Alto Minho, Viana do Castelo, PRT; 3 Critical Care, Unidade Local de Saude do Alto Minho, Viana do Castelo, PRT; 4 Pathology, Unidade Local de Saúde do Alto Minho, Viana do Castelo, PRT

**Keywords:** acute febrile neutrophilic dermatosis, corticosteroid therapy, neutrophilic dermatoses, selective iga deficiency, sweet's syndrome

## Abstract

Sweet's syndrome is an acute febrile neutrophilic dermatosis characterised by fever, neutrophilia, and painful cutaneous lesions.

We describe a case of a 31-year-old male admitted with a two-week history of fever up to 39°C and painful erythematous papules, scattered and coalescing into plaques over the trunk and limbs, together with pustules on an erythematous base over the palms and dorsum of the hands. No relevant epidemiological exposure or recent drug exposure was identified. Investigation showed an erythrocyte sedimentation rate of 40 mm/h, leucocytosis of 14,380/µL with 77% neutrophils and no eosinophilia, and a C-reactive protein of 1.21 mg/dL. Liver and renal function, albumin, lactate dehydrogenase, serum iron, ferritin, and serum protein electrophoresis were normal, and a respiratory viral panel was negative. Blood, urine, and pustule cultures, an interferon-gamma release assay, and serology for human immunodeficiency virus, syphilis, hepatitis A, B, C, and E viruses, cytomegalovirus, and Epstein-Barr virus were negative, as was an autoantibody panel. Serum immunoglobulin A was below the detection limit (<0.07 g/L) before corticosteroid therapy and on all repeat measurements, including after treatment had been discontinued, with normal immunoglobulin G and M, and IgG-based coeliac serology was negative. Computed tomography of the chest, abdomen, and pelvis and peripheral blood immunophenotyping showed no abnormalities. A skin biopsy demonstrated intraepidermal blisters and a superficial perivascular neutrophilic infiltrate without vasculitis, spongiform pustules, or psoriasiform changes. The clinical and laboratory criteria for classical Sweet's syndrome were fulfilled, and the histology was compatible with, although not classical for, the syndrome. Prednisolone 60 mg/day (approximately 0.6 mg/kg/day) was started on clinical suspicion of the syndrome. After one week, the fever had resolved, but the lesions worsened slightly, prompting escalation to 100 mg/day (approximately 1 mg/kg/day), after which they regressed progressively and resolved completely within three weeks of dose escalation, with normalisation of the leucocyte count. Corticosteroids were tapered and discontinued before the three-month assessment, at which there was no relapse and no evidence of malignancy.

This case illustrates three practical points: an acral pustular morphology does not exclude the diagnosis; early worsening under corticosteroid therapy may reflect an initial dose insufficient for body weight rather than an incorrect diagnosis, once infection has been rigorously excluded; and the coexisting finding of selective immunoglobulin A deficiency carries independent implications for coeliac disease screening and long-term follow-up.

## Introduction

In 1964, Robert Douglas Sweet described eight women with fever, peripheral neutrophilia, and tender erythematous plaques underpinned by a dense dermal infiltrate of mature neutrophils, and named the condition acute febrile neutrophilic dermatosis [1]. The eponym Sweet's syndrome (SS) came into use shortly afterwards. The disorder is now regarded as the prototype of the neutrophilic dermatoses, a group united by neutrophil-rich cutaneous infiltration in the absence of infection, by the occurrence of overlapping and transitional forms, and by a shared tendency to accompany systemic disease [2].

Diagnosis rests on the criteria of Su and Liu [3], as modified by von den Driesch [4]. Both major criteria must be present: the abrupt onset of tender erythematous plaques or nodules, and a dermal neutrophilic infiltrate without leucocytoclastic vasculitis. At least two of four minor criteria are also required, namely, fever above 38°C; an association with malignancy, inflammatory disease, or pregnancy, or a preceding upper respiratory or gastrointestinal infection or vaccination; a prompt response to systemic corticosteroids or potassium iodide; and abnormal laboratory values at presentation, defined as at least three of an erythrocyte sedimentation rate above 20 mm/h, a raised C-reactive protein, a leucocyte count above 8000/µL, and more than 70% neutrophils.

By aetiology, SS is conventionally divided into classical or idiopathic disease, malignancy-associated disease, drug-induced disease, disease accompanying systemic inflammatory disorders, and pregnancy-associated disease [5]. Classical disease accounts for most cases, shows a marked female predominance, and peaks between the third and sixth decades, with recurrence in roughly a third of patients [5]. Around a fifth of all patients prove to have an underlying haematological or solid malignancy, acute myeloid leukaemia being the single most common [6], and the eruption may precede the tumour by several years [7]. Paediatric presentations are rare, and associated malignancy has also been reported in children [8,9]. Patients with malignancy-associated disease present at an older age than those with classical disease [10]. Among drugs, granulocyte colony-stimulating factor is implicated most often, with antibiotics, antiepileptics, antihypertensives, antiretrovirals, antineoplastic agents, and oral contraceptives among the many others reported; drug-induced disease characteristically appears about two weeks after first exposure and recurs on rechallenge [5].

The eruption begins abruptly, often accompanied by malaise, myalgia, arthralgia, and headache. Lesions are tender rather than pruritic and consist of erythematous to violaceous papules and nodules that enlarge and coalesce into asymmetrical plaques, classically on the face, neck, and upper limbs [11]. Marked dermal oedema may give the surface a deceptively blistered appearance, the so-called pseudo-vesicle. Genuine vesicles, bullae, and ulceration occur mainly in malignancy-associated disease and can be difficult to distinguish from pyoderma gangrenosum [12,13]. A pustular variant, in which pustules arise on an erythematous base and involvement is predominantly acral, was formerly reported under the names neutrophilic dermatosis of the dorsal hands and pustular vasculitis of the dorsal hands [11,12].

Leucocytosis with neutrophilia and a raised erythrocyte sedimentation rate are the most consistent laboratory abnormalities, usually alongside elevation of other acute-phase reactants [5]. The characteristic histological picture is a dense infiltrate of mature neutrophils in the superficial dermis with marked papillary oedema and nuclear dust, sparing of the epidermis and no true vasculitis, although lymphocytes, eosinophils, and histiocytes may also be present [14].

Roughly 15% of cases arise in the setting of a systemic inflammatory disorder, most often inflammatory bowel disease [15]. Less firmly established associations have been described with infection by the human immunodeficiency virus, tuberculosis, autoimmune disease, and primary immunodeficiency. The pathogenesis remains incompletely understood; dysregulated innate immunity, with inflammasome activation and cytokine-driven neutrophil recruitment, is the prevailing explanation, and SS is increasingly placed among the autoinflammatory disorders [14].

We report a case of classical SS in a 31-year-old male with three features worth documenting: a pustular component involving the palms and dorsum of the hands, an acral distribution distinct from the classic pattern; a transient worsening of the lesions after corticosteroid therapy was started, which prompted dose escalation rather than a change of diagnosis; and the incidental discovery of selective immunoglobulin A deficiency.

## Case presentation

A 31-year-old male with a personal history of hypersensitivity to salicylates and non-steroidal anti-inflammatory drugs, followed in an immunoallergology outpatient clinic, with no other significant comorbidity and whose only regular medication was an oral antihistamine, was admitted with a two-week history of fever up to 39°C and scattered painful erythematous papular lesions coalescing into plaques, predominantly over the trunk and limbs, together with pustules on an erythematous base over the palms and dorsum of the hands (Figure 1).

**Figure 1:**
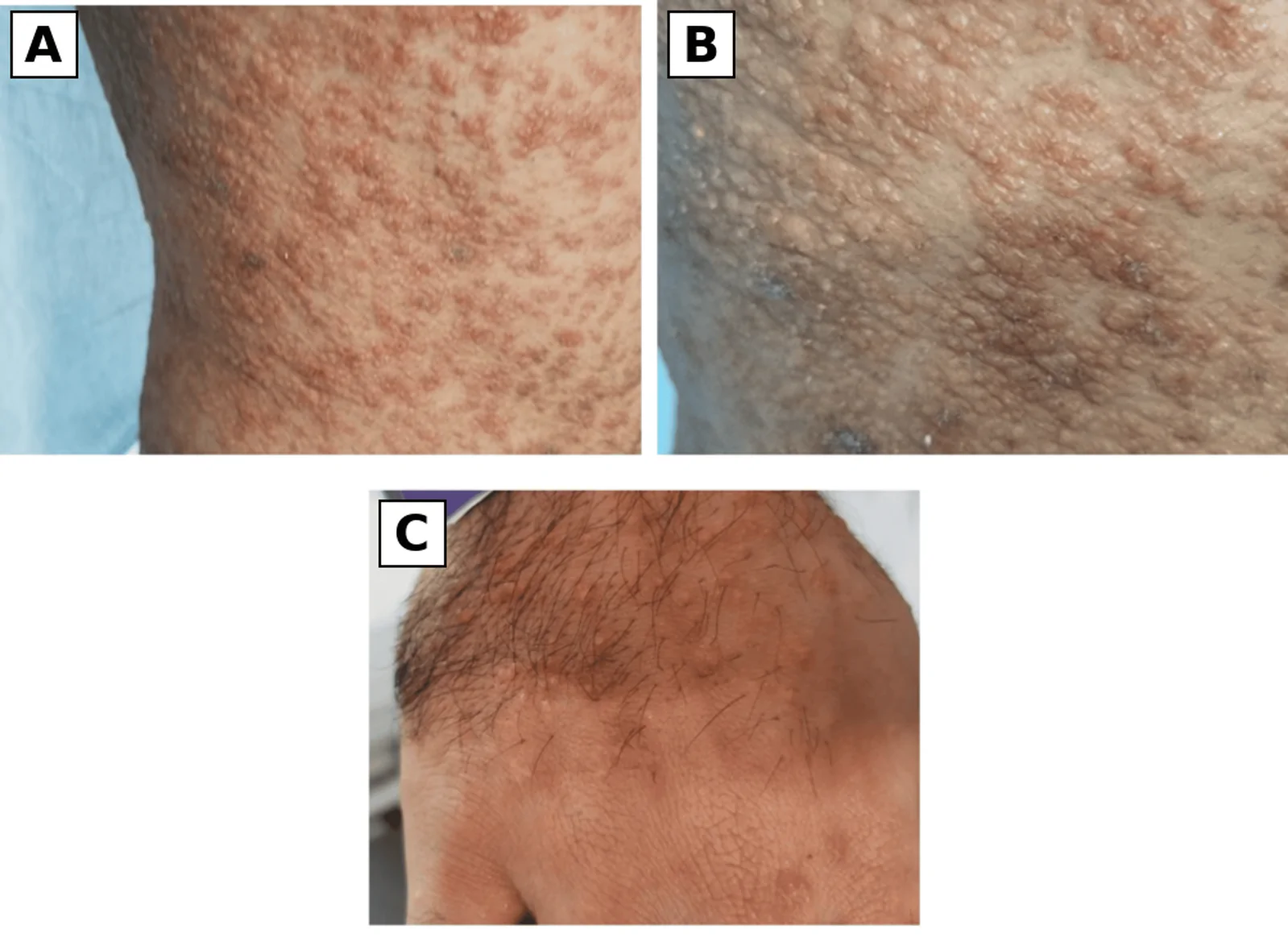
Cutaneous lesions at presentation. (A) Lateral aspect of the trunk, showing numerous erythematous papules, several coalescing into ill-defined plaques. (B) Close-up of the trunk, in which the papules are monomorphic, and several are surmounted by small pustules. (C) Dorsum of the hand, showing erythematous papules and pustules over the dorsal surface and the first web space.

He reported no gastrointestinal complaints, specifically no diarrhoea, vomiting, or abdominal pain, and no recent respiratory or urinary tract infection. He denied weight loss, excessive sweating, lymphadenopathy, fatigue, odynophagia, jaundice, and cough. There was no personal or family history of autoimmune disease, and no relevant epidemiological exposure or recent drug administration was identified.

Initial physical examination revealed painful erythematous papular lesions coalescing into plaques, predominantly over the trunk and limbs, and pustules over the palms and dorsum of the hands. The nails, scalp, and oral and genital mucosae were examined and were normal, with no nail pitting or onycholysis. Cardiac and pulmonary auscultation was normal, and there were no signs of hepatomegaly, splenomegaly, or ascites. No peripheral lymphadenopathy was palpable.

Laboratory investigations at admission are summarised in Table 1. They showed an elevated erythrocyte sedimentation rate, leucocytosis with neutrophilia and no eosinophilia, and a raised C-reactive protein. Haemoglobin (14.2 g/dL) and platelet count (276 000/µL) were within normal limits, and the peripheral blood smear showed no morphological abnormalities. Liver function tests, albumin, lactate dehydrogenase, serum iron, ferritin, serum protein electrophoresis, and renal function tests were normal, and a respiratory viral panel was negative.

**Table 1:** Laboratory investigations at admission.

Laboratory parameter	Result at admission	Reference range
Haemoglobin (g/dL)	14.2	13.0-17.0
Platelets (/µL)	276,000	150,000-400,000
Leucocytes (/µL)	14,380	4000-11,000
Neutrophils (%)	77	40-70
Eosinophils (%)	1.5	1-4
Peripheral blood smear	No morphological abnormalities	-
Erythrocyte sedimentation rate (mm/h)	40	0-20
C-reactive protein (mg/dL)	1.21	<0.5
Alanine aminotransferase (U/L)	36	<41
Aspartate aminotransferase (U/L)	33	<40
Alkaline phosphatase (U/L)	58	40-130
Gamma-glutamyl transpeptidase (U/L)	57	8-61
Total bilirubin (mg/dL)	0.6	0.2-1.2
Albumin (g/dL)	4.0	3.5-5.2
Lactate dehydrogenase (U/L)	139	135-225
Serum iron (µg/dL)	64	59-158
Ferritin (ng/mL)	150	30-400
Creatinine (mg/dL)	0.8	0.7-1.2
Urea (mg/dL)	31	17-43
Serum protein electrophoresis	Normal pattern, no monoclonal band	Normal pattern
Immunoglobulin A (g/L)	<0.07 (below the detection limit; confirmed on repeat testing)	0.7-4.0
Immunoglobulin G (g/L)	15.0	7.0-16.0
Immunoglobulin M (g/L)	2.1	0.4-2.3
Respiratory viral panel	Negative	Negative

Serology for human immunodeficiency virus was negative. Viral causes of hepatitis were excluded, with negative serologies for hepatitis A, B, C, and E viruses, cytomegalovirus, and Epstein-Barr virus. Blood cultures and urine culture were sterile, and bacterial culture of pustule content was negative. An interferon-gamma release assay and syphilis serology were also negative. Antinuclear antibodies, antineutrophil cytoplasmic antibodies, anti-DNA antibodies, and anti-SSA and anti-SSB antibodies were all negative, and the C3 and C4 fractions of complement were within normal limits.

An isolated immunoglobulin A deficiency was noted. Serum immunoglobulin A was below the laboratory's lower limit of detection (<0.07 g/L) at admission, before corticosteroid therapy was started, and on repeat testing on days 7 and 14, with normal immunoglobulin G and M (Table 1). It remained undetectable on a further measurement after corticosteroid therapy had been discontinued. As immunoglobulin A-based serological assays are unreliable in this setting, coeliac disease screening was performed with IgG-class antibodies against tissue transglutaminase, deamidated gliadin peptides, and endomysium, which were negative.

The remainder of the aetiological study, covering infectious, autoimmune, and neoplastic causes, was negative. Computed tomography of the chest, abdomen, and pelvis and peripheral blood immunophenotyping showed no abnormalities. Following multidisciplinary discussion, bone marrow aspiration and biopsy were not performed, as the comprehensive work-up, including peripheral blood immunophenotyping, showed no abnormalities suggestive of haematological disease.

A skin biopsy of a lesion on the abdomen was performed, and corticosteroid therapy with prednisolone 60 mg/day (approximately 0.6 mg/kg/day; body weight 95 kg) was initiated on suspicion of SS. Histological examination demonstrated intraepidermal blisters and a superficial perivascular neutrophilic infiltrate, with no signs of vasculitis (Figures 2-4). There were no subcorneal or spongiform pustules, psoriasiform epidermal hyperplasia, or parakeratosis. The infiltrate did not have the dense, diffuse dermal distribution of classical SS, but was considered compatible with the diagnosis in this clinical context.

**Figure 2:**
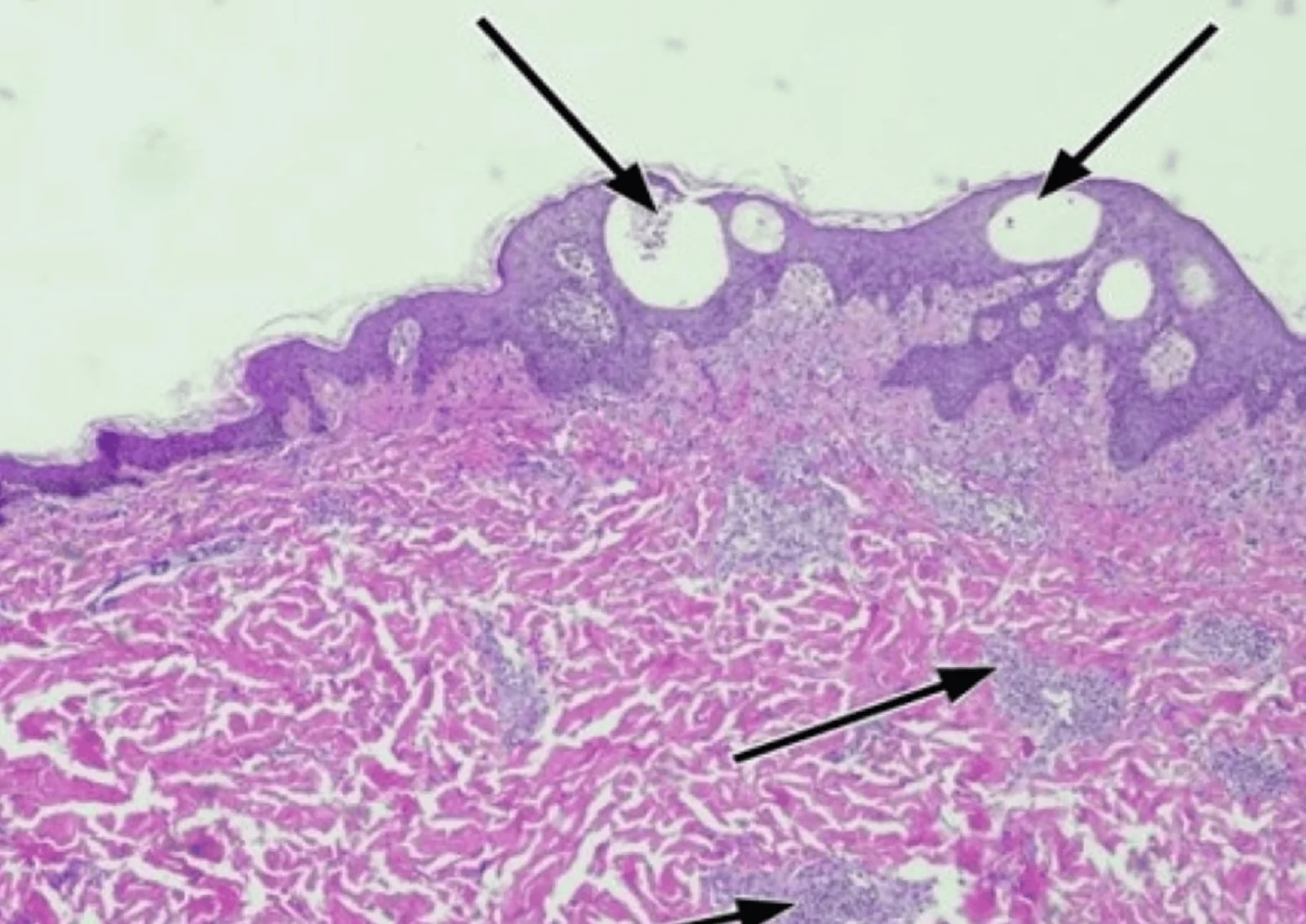
Incisional (wedge) skin biopsy showing intraepidermal blisters and a superficial perivascular infiltrate. Black arrows indicate the intraepidermal blisters within the epidermis (upper field) and the perivascular inflammatory infiltrate in the superficial dermis (lower field). Haematoxylin and eosin.

**Figure 3:**
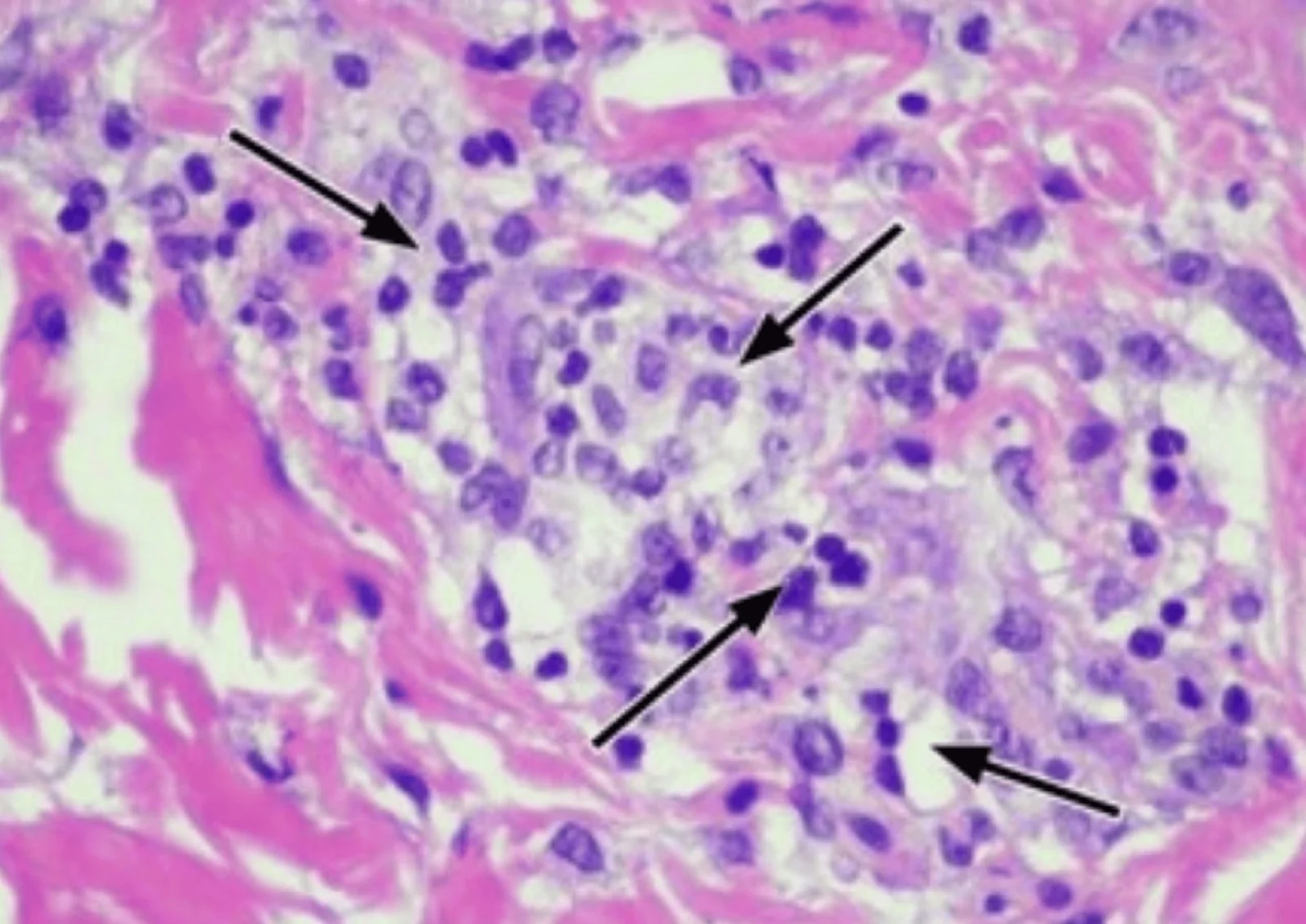
Details of the superficial perivascular infiltration with neutrophils and occasional eosinophils. Black arrows indicate representative polymorphonuclear neutrophils with segmented nuclei within the perivascular infiltrate. Haematoxylin and eosin.

**Figure 4:**
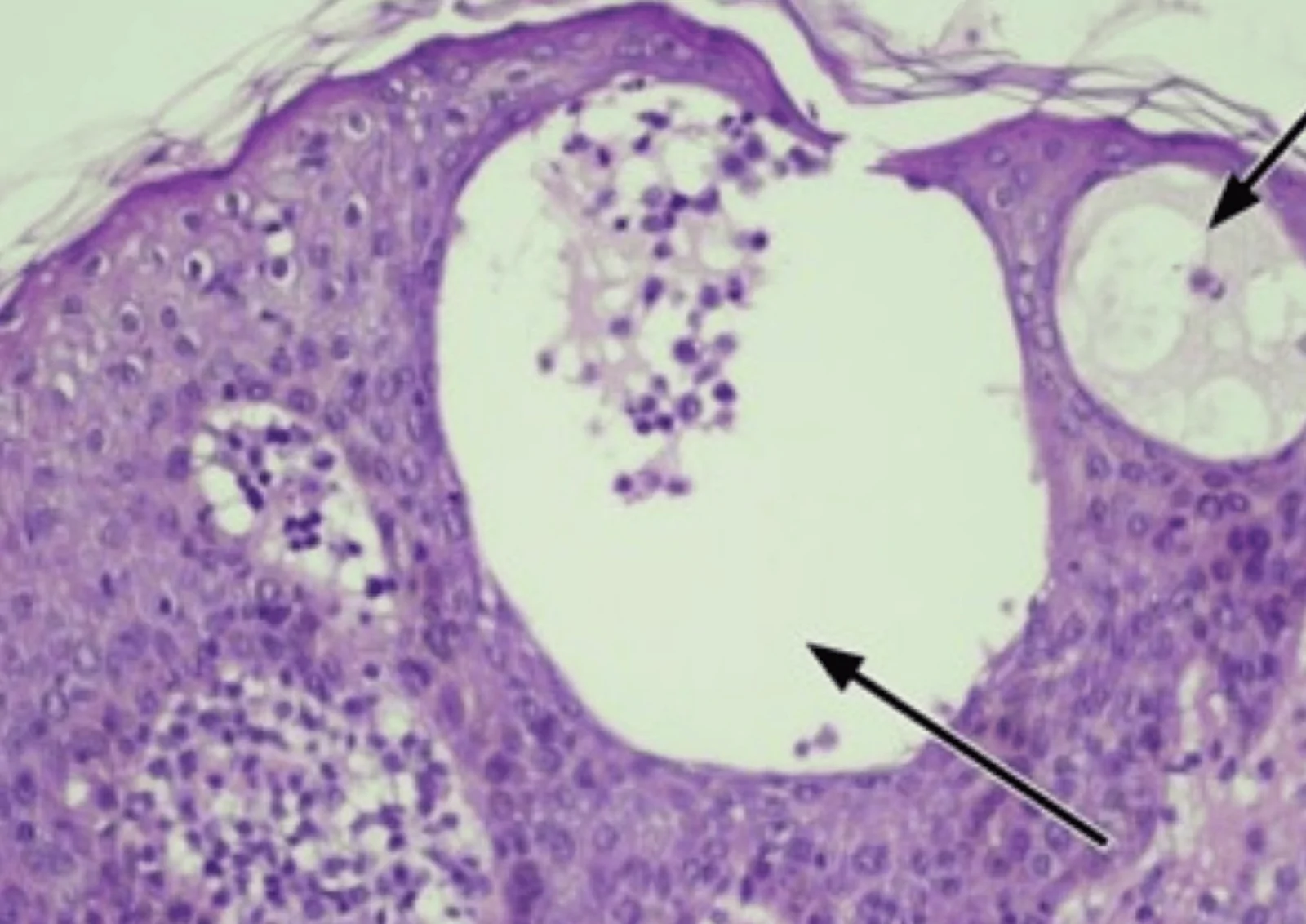
Details of the intraepidermal blisters. Black arrows indicate two intraepidermal blister cavities, the larger of which contains neutrophils. Haematoxylin and eosin.

After one week of treatment, the patient had become afebrile, but a mild worsening of the cutaneous lesions was observed. Following reassessment for an occult infection and for pathergy, both of which were considered unlikely, the dose of prednisolone was increased to 100 mg/day (approximately 1 mg/kg/day), followed by progressive regression and complete resolution of the lesions within three weeks of dose escalation.

Resolution of the leucocytosis with neutrophilia was confirmed by laboratory testing. Fever resolved by day 7, before dose escalation, and did not recur, whereas the cutaneous lesions resolved gradually and completely within three weeks of dose escalation. Follow-up photographs of the resolved lesions were not obtained.

Once regression of the lesions was evident, after one week at 100 mg/day, prednisolone was tapered by 10-20 mg per week and discontinued before the three-month assessment, with no flare or relapse during tapering. During corticosteroid therapy, capillary blood glucose and blood pressure were monitored; glucose rose slightly without requiring treatment, and blood pressure remained below 140 mmHg. Gastric protection with pantoprazole and bone protection with calcium 1200 mg/day and vitamin D 1000 IU/day were given, and no other adverse effects were observed. Follow-up continued in the internal medicine and dermatology outpatient clinics. The clinical course is summarised in Table 2.

**Table 2:** Timeline of the clinical course.

Time point	Event
Day −14	Onset of fever and skin lesions
Day 0	Admission; skin biopsy (abdomen); serum IgA <0.07 g/L; prednisolone 60 mg/day (≈0.6 mg/kg/day) started
Day 7	Afebrile; mild worsening of lesions; reassessment for infection and pathergy; prednisolone increased to 100 mg/day (≈1 mg/kg/day)
Day 14	Lesions regressing; tapering by 10-20 mg/week started
Day 28 (approx.)	Complete resolution of skin lesions
Before 3 months	Corticosteroids discontinued, without flare; serum IgA still <0.07 g/L
3 months	Last follow-up; no relapse and no evidence of malignancy

## Discussion

The clinical and laboratory features of this case fulfilled the criteria of Su and Liu, as modified by von den Driesch, with one important qualification [3,4]. The abrupt onset of painful erythematous plaques satisfied the first major criterion. The second major criterion requires a dense neutrophilic dermal infiltrate without leucocytoclastic vasculitis; the biopsy showed a neutrophil-predominant infiltrate without vasculitis, but in a superficial perivascular rather than a dense, diffuse distribution, so this criterion was only partially met. Pyrexia exceeding 38°C and abnormal laboratory values at presentation (erythrocyte sedimentation rate above 20 mm/h, raised C-reactive protein, leucocyte count above 8000/µL and more than 70% neutrophils) accounted for two of the minor criteria. The response to systemic corticosteroid therapy, although ultimately complete, was not prompt, since the lesions initially worsened and required dose escalation; this criterion was therefore not counted. The remaining minor criterion, an association with underlying malignancy, inflammatory disease, or pregnancy or a preceding respiratory or gastrointestinal infection, was not met. The diagnosis of classical, idiopathic SS, in its pustular variant, was therefore based on the combination of the clinical picture, the laboratory findings, compatible histology, the clinical course, and the systematic exclusion of alternative diagnoses, rather than on strict fulfilment of the classification criteria.

The histological pattern deserves comment, since it departed from the textbook description. Rather than a dense, diffuse neutrophilic infiltrate filling the superficial dermis, the biopsy showed intraepidermal blistering with a superficial perivascular neutrophilic infiltrate. This distribution is consistent with the pustular variant, in which neutrophils accumulate intraepidermally to form the clinically visible pustules, and the dermal infiltrate was neutrophil-predominant without leucocytoclastic vasculitis. Occasional eosinophils were present within the infiltrate, an accepted finding in SS that should not be confused with peripheral blood eosinophilia, which was absent in this patient. The presence of genuine intraepidermal blistering, which has been associated more often with malignancy-associated disease [12,13], reinforced the need for the extended neoplastic work-up described above. We acknowledge that intraepidermal neutrophilic collections with occasional eosinophils may also be seen in acute generalised exanthematous pustulosis; the histological findings are therefore supportive rather than specific, and the diagnosis rests on the combination of clinical, laboratory, and histological features, the course of the disease, and the exclusion of alternative diagnoses.

Drug-induced SS was considered improbable. The patient was taking only antihistamines and no agent had been introduced in the preceding weeks. The documented hypersensitivity to salicylates and non-steroidal anti-inflammatory drugs merits comment, since these agents are recognised precipitants, but he had systematically avoided them and the temporal relationship required for a diagnosis of drug-induced disease was absent [5].

One feature of this presentation deserves emphasis: the eruption included pustules on an erythematous base over the palms and dorsum of the hands. Acral pustular involvement of this kind is characteristic of the pustular variant, described historically under the names neutrophilic dermatosis of the dorsal hands and pustular vasculitis of the dorsal hands [11,12]. Recognising this variant matters clinically, because acral pustulosis may be readily attributed to infection or to a pustular drug eruption, with potential diagnostic delay and unnecessary antimicrobial exposure.

In a febrile patient with an acute papulopustular eruption and neutrophilia, the main differential diagnoses were acute generalised exanthematous pustulosis, generalised pustular psoriasis, leucocytoclastic vasculitis, pyoderma gangrenosum, Behçet's disease, disseminated bacterial, mycobacterial or fungal infection, and erythema multiforme or Sweet-like drug eruptions [12]. Acute generalised exanthematous pustulosis was the main alternative, given the pustular component and the intraepidermal neutrophilic collections on biopsy. It was considered unlikely because no new drug had been introduced before the onset of the eruption, and because the clinical course did not fit: acute generalised exanthematous pustulosis typically begins within days of drug exposure and resolves spontaneously within about two weeks, whereas in this patient, fever and skin lesions had persisted for two weeks before admission and continued to progress until corticosteroid escalation [16]. The predominance of painful erythematous papules coalescing into plaques, rather than diffuse oedematous erythema studded with pinhead pustules, also favoured SS. Generalised pustular psoriasis required particular consideration, because it may present de novo without a personal or family history of psoriasis, combines fever with neutrophilia and raised inflammatory markers, and is classically precipitated by the withdrawal of systemic corticosteroids. Several features argued against it. The eruption consisted of painful papules coalescing into plaques rather than sheets of erythema studded with sterile pustules; the nails, scalp, and mucosae were normal, with no nail pitting or onycholysis; the biopsy showed no subcorneal or spongiform pustules of Kogoj, psoriasiform epidermal hyperplasia or parakeratosis; and corticosteroids were tapered and discontinued without any flare, with no recurrence at three months. We acknowledge that a single biopsy cannot exclude it with complete certainty, and dermatological follow-up continues. Vasculitis and pyoderma gangrenosum were excluded on histological grounds and by the absence of ulceration or pathergy, and Behçet's disease by the absence of aphthosis, ocular involvement, and typical systemic features.

Infection was excluded by an extensive negative microbiological work-up, including a respiratory viral panel, blood cultures, urine culture, culture of pustule content, an interferon-gamma release assay, and syphilis serology, and the eruption resolved under immunosuppression alone. SS remains in practice a diagnosis of exclusion, and infection must be confidently ruled out before systemic corticosteroids are introduced.

A distinctive feature of this case was the mild worsening of the cutaneous lesions after one week of prednisolone 60 mg/day, although the fever had already resolved. This prompted a systematic reassessment for an unrecognised infectious process, since corticosteroids may unmask or aggravate infection, and for pathergy, given that SS lesions may be precipitated by trauma, including at the biopsy site. Both were considered unlikely. In retrospect, the initial dose corresponded to only about 0.6 mg/kg/day in a 95 kg patient, below the usual starting dose of about 1 mg/kg/day [5], and the phenomenon was interpreted as insufficient initial suppression of an active neutrophilic process. Escalation to 100 mg/day therefore brought the dose to approximately 1 mg/kg/day, the standard dose, rather than to a high-dose regimen. Oral escalation was preferred to intravenous methylprednisolone pulses as the treating team's individual decision, because the patient remained clinically stable, with no extracutaneous or organ involvement, was tolerating oral therapy, and had just been reassessed for an occult infection. Escalation was followed by complete resolution within three weeks, and tapering was completed without relapse. Early clinical deterioration should therefore not by itself overturn the diagnosis, provided infection has been rigorously excluded and the dose is adequate for body weight.

Systemic corticosteroids remain the treatment of choice. Constitutional symptoms typically settle within a day or two of starting treatment, with the skin lesions following over the subsequent one to two weeks. Treatment usually begins at around 1 mg/kg/day of prednisolone as a single morning dose, reduced to about 10 mg/day over four to six weeks, although a minority of patients need two to three months of therapy [5]. Tapering too quickly is a recognised precipitant of relapse. For patients who relapse during withdrawal, who need prolonged treatment, or in whom corticosteroids are contraindicated, potassium iodide, colchicine, and dapsone are the usual alternatives, while ciclosporin, indometacin, clofazimine, and, more recently, agents targeting tumour necrosis factor-α or interleukin-1 have been used in refractory disease [12,14]. In drug-induced disease, withdrawal of the offending agent is the essential therapeutic step.

Selective immunoglobulin A deficiency (SIgAD) is the most common primary immunodeficiency, defined by a serum immunoglobulin A concentration below 0.07 g/L with normal immunoglobulin G and M in a patient over four years of age, once other causes of hypogammaglobulinaemia have been excluded [17]. Most affected individuals never come to medical attention. Any link with neutrophilic dermatosis is speculative and has not been established. It could be hypothesised that loss of secretory immunoglobulin A weakens mucosal immune exclusion, increasing the antigenic load that reaches the systemic compartment, and that the immune dysregulation described in SIgAD favours the cytokine-driven neutrophil recruitment that represents the final common pathway of the neutrophilic dermatoses [14,18], but this remains conjecture.

Given the relatively high population prevalence of SIgAD, a coincidental association is at least as likely, and we regard the finding as a coexisting condition with independent clinical implications rather than as a cause of, or predisposing factor for, SS. In this patient, serum immunoglobulin A was below the detection limit (<0.07 g/L) before corticosteroid therapy, on days 7 and 14, and again after corticosteroid therapy had been discontinued, which argues against a transient effect of the acute illness or of treatment. Secondary causes of hypogammaglobulinaemia were considered unlikely: serum albumin and immunoglobulin G and M levels were normal, arguing against protein loss, and he was not taking any medication known to lower immunoglobulin A levels.

The identification of SIgAD nevertheless carries practical consequences beyond the aetiological discussion. Screening for coeliac disease is recommended, both because of the recognised association and because immunoglobulin A-based serological assays are unreliable in this setting, so that IgG-based assays must be used, as was done in this patient. Long-term surveillance for the emergence of autoimmune disease and, in a minority of patients, for progression to common variable immunodeficiency is also warranted [17,18].

Prognosis is favourable. Left alone, the eruption may run a protracted course over several months, whereas treated patients typically clear within days to a few weeks, and the lesions heal without leaving scars. Relapse is nonetheless common, affecting roughly one patient in three with classical disease [5,19]. Two considerations therefore shape follow-up in a patient such as ours. First, because the dermatosis can be the presenting sign of an occult tumour and may antedate it by many years [6,7], an initial search for neoplasia and continued vigilance thereafter are both justified even when, as here, the immediate work-up is negative. In the absence of cytopenias or other haematological abnormalities, bone marrow examination was deferred after multidisciplinary discussion, with the option of reconsidering it should haematological changes emerge during follow-up. Second, SIgAD requires monitoring in its own right. Our patient remains under regular review in the departments of internal medicine and dermatology, and neither relapse nor malignancy has emerged after three months of follow-up.

## Conclusions

SS should be considered in any patient presenting with fever, neutrophilia, and painful erythematous cutaneous lesions once infection has been excluded. This case illustrates three points of practical relevance. First, the diagnosis should not be discarded on the basis of an atypical, acral pustular morphology or of histology that falls short of the classical dense infiltrate, provided alternative diagnoses, notably generalised pustular psoriasis and acute generalised exanthematous pustulosis, have been carefully excluded. Second, an initial worsening under corticosteroid therapy does not by itself invalidate the diagnosis and should prompt a review of whether the dose is adequate for body weight, provided infection has been rigorously excluded. Third, selective immunoglobulin A deficiency, a coexisting finding whose relationship to the syndrome is uncertain, carries independent implications for coeliac disease screening and long-term follow-up. Given the recognised association of the syndrome with malignancy and autoimmune disease, structured and sustained surveillance is warranted.
